# Deletion of MicroRNA-21 Impairs Neovascularization Following Limb Ischemia: From Bedside to Bench

**DOI:** 10.3389/fcvm.2022.826478

**Published:** 2022-04-26

**Authors:** Wei-Ting Chang, Yu-Wen Lin, Po-Sen Huang, You-Cheng Lin, Shih-Ya Tseng, Ting-Hsing Chao, Zhih-Cherng Chen, Jhih-Yuan Shih, Chon-Seng Hong

**Affiliations:** ^1^Division of Cardiology, Department of Internal Medicine, Chi-Mei Medical Center, Tainan, Taiwan; ^2^Department of Biotechnology, Southern Taiwan University of Science and Technology, Tainan, Taiwan; ^3^College of Medicine, Institute of Clinical Medicine, National Cheng Kung University, Tainan, Taiwan; ^4^Department of Internal Medicine, National Cheng Kung University College of Medicine and Hospital, Tainan, Taiwan; ^5^Division of Cardiology, Department of Internal Medicine, National Cheng Kung University Hospital, Tainan, Taiwan

**Keywords:** miR-21, limb ischemia, male, angiogenesis, translational study

## Abstract

With an increasing prevalence, peripheral arterial disease (PAD), cause by atherosclerosis is a new threat to public health beyond coronary artery disease and involves aberrant vascular endothelial cell proliferation and angiogenesis. The degree of vascular remodeling is influenced by the processes described. MicroRNA-21 (miR-21) has been found to play a critical role in cellular functions, including angiogenesis. Nevertheless, the effect of miR-21 on endothelial cells in response to hypoxia is largely unknown. Using wild-type C57BL/6J and miR-21^–/–^ mice, we compared the capability of angiogenesis in response to hindlimb hypoxic/ischemia. *In an in vitro* study, we further studied whether overexpression of miR-21 mitigates hypoxia-induced apoptosis and impaired angiogenesis. Also, we prospectively collected the sera of patients with limb ischemia and followed the clinical information, including major adverse limb events (MALEs). Using laser Doppler perfusion imaging and CD31 staining, compared with miR-21^–/–^ mice, wild-type mice expressed a significantly higher capability of angiogenesis and less apoptosis following 28 days of hindlimb hypoxic/ischemic surgery. In our *in vitro* study, after 24 h of hypoxia, proliferation, migration, and tube formation were significantly impaired in cells treated with the miR-21 inhibitor but rescued by the miR-21 mimic. Mechanistically, by suppressing PTEN/PI3K/AKT, miR-21 promoted angiogenesis and suppressed apoptosis in endothelial cells post hypoxia. In patients with limb ischemia, the high expression of circulating miR-21 was associated with less subsequent MALE. Collectively, miR-21 could be a biomarker associated with the endogenous ability of angiogenesis and reflect subsequent MALE in patients. Additionally, abolishing miR-21 impairs angiogenesis and promotes apoptosis post limb ischemia. Further studies are required to elucidate the clinical applications of miR-21.

## Introduction

With its soaring prevalence, peripheral artery disease (PAD) has been catching attention (1). In a systematic review including 118 articles, at patient ages of 40–44 years, the prevalence of PAD was approximately 4.32%, while at ages of 80–84 years, the prevalence increased to 21.24% (1). Regarding the treatment of PAD, despite improvements in surgical and endovascular interventions, the patency of limb vessels is limited, and some patients at old age or with comorbidities may not be optimal candidates (2). Anti-platelet agents increase walking distance in patients with PAD but fail to reduce major adverse cardiovascular events (3, 4). Although previous clinical trials showed that a combination of novel oral anticoagulants (NOACs) with traditional antiplatelet therapy may produce promising results in reducing major adverse limb events (MALEs), there are still safety issues with bleeding (5, 6). Additionally, similar to but still different from coronary arterial disease, PAD involves not only the development of atherosclerosis but also aberrant vascular endothelial cell proliferation and angiogenesis (7, 8). Angiogenesis is the growth and proliferation of blood vessels from the existing vasculature, which increases the microvascular density of the occluded arteries that impair perfusion (8). However, most trials of angiogenic factors and cell therapies for PAD failed. Therefore, to date, an efficient pharmacological therapy is lacking.

MicroRNAs (miRNAs) are endogenous non-coding small RNA molecules (20–25 nucleotides) that regulate a wide range of physiological and pathological processes, including cell development, metabolism, aging, and death (9, 10). Among multiple miRNAs, miRNA−21 (miR−21) has been reported to promote angiogenesis and to suppress apoptosis (11). Nevertheless, the effect of miR-21 on endothelial cells in response to a hypoxic environment is largely unknown. Herein, we aimed to investigate the regulatory mechanism and therapeutic potential of miR-21 in the treatment of PAD.

## Materials and Methods

### Animal Model of Critical Hindlimb Ischemia

All animal experiments were approved and conducted in accordance with the strict guidelines of the Subcommittee on Research Animal Care of Chi-Mei Medical Center, and the standards met the Guide for the Care and Use of Laboratory Animals. Twelve-week-old adult male C57BL/6J mice were anesthetized with an intraperitoneal injection of pentobarbital (80 mg/kg) and received unilateral femoral artery ligation and excision as described previously. After arterial ligation, mice were immediately assigned to the following experimental groups: wild-type (WT) sham, WT ischemia, miR-21^–/–^ sham, and miR-21^–/–^ ischemia. After surgery, blood flow in the ischemic and contralateral non-ischemic limbs was measured using a serial laser Doppler imaging system (PeriScan PIM 3 Systems; Perimed AB, Sweden) on Days 0, 7, 14, 21, and 28. The perfusion index was determined as the ratio of ischemic to non-ischemic hindlimb blood flow for each mouse. After the end of the experiment, calf muscles were harvested, and the weight loss of mass in the ischemic muscle was expressed as ischemic (left) calf muscle/non-ischemic (right) calf muscle.

### Capillary Density Analysis and MicroRNA-21 *in situ* Hybridization

Mice were sacrificed at 28 days after hindlimb ischemia and calf muscles were obtained from the control (right) and ischemic side (left) and then fixed in 4% paraformaldehyde and embedded in paraffin. The tissue sections were stained with anti-CD31 antibody (1:100, Abcam, Cambridge, MA, United States), followed by incubation with anti-rabbit secondary antibody (1:400, Abcam, Cambridge, MA, United States). The images were captured by a fluorescence microscope (Olympus BX51, Olympus Optical Co., Ltd., Tokyo, Japan). Capillary density was quantified by counting the mean number of capillaries, as revealed by positive expression of CD31 on endothelial cells. The miR-21 oligonucleotide probe was designed and purchased from Li-Tzung biotechnology (Kaohsiung, Taiwan). The staining method has been described previously (12, 13).

### Cell Culture and *in vitro* Transfection

HUVECs were kind from Dr. Ching-Ping Chang (Chi-Mei Medical Center, Tainan, Taiwan) and cultured in standard endothelial cell growth medium (Gibco, Thermo Fisher Scientific, Massachusetts, United States) with 10% fetal bovine serum. HUVECs were exposed to hypoxia (0.1% oxygen; BioSpherix Medical) to mimic the HUVECs under ischemic condition in hindlimb ischemia model. For *In vitro* transfection, the miRNA mimics or inhibitors was used to overexpression or knockdown miR-21 expression in HUVECs. Briefly, TransIT-X2^®^ Transfection Reagent (Mirus Bio, Madison, United States) was used to transfect the miR-21 mimic (5′-uagcuuaucagacugauguuga-3′), miR-21 inhibitor (5′-ucaacaucagucugauaagcua-3′) or scrambled control (QIAGEN, Hilden, Germany) into HUVECs for 24 h at 37^°^C, the concentration used for miR-21 mimic or inhibitor transfection was 5 and 10 nM, respectively.

### RNA Isolation and Quantitative RT-PCR

Total RNAs were isolated with Trizol reagent (Ambion) as described by the manufacturer. cDNA was generated using the Taqman MicroRNA Assays (Foster City, CA). Primer sequence used for miR-21-5p reverse real time polymerase chain reaction (PCR) is as listed: uagcuuaucagacugauguugac (Life Technology, Carlsbad, CA). The mRNA levels of miR-21 were measured using 7500 Fast Real-Time PCR system (Applied Biosystems, Foster City, CA, United States). AS previously described (14), for intra-cellular microRNA expression, the level of miRNA was normalized to U6 while for circulating microRNA expression the level of miRNA was normalized to spike in RNA. The final fold expression changes were calculated using the equation 2^–Δ^ ^Δ^ *^Ct^*.

### Bromodeoxyuridine Cell Proliferation Assay

The cell proliferation was measured using a Bromodeoxyuridine (BrdU) kit (Invitrogen; Carlsbad, CA, United States) according to the manufacturer’s protocol and quantitated on a microplate reader. Briefly, Total of 2 × 10^5^ cells of HUVECs were seeded into wells and transfected with miR-21 mimic (5 nM), inhibitor (10 nM), and their respective negative control, allowed to grow for 24 h at 37^°^C under hypoxia (3% oxygen; BioSpherix Medical). After incubation, the cell proliferation was determined using a spectrophotometer (MULTISKAN. GO, Thermo Fisher Scientific, CA, United States) with a wavelength set at 450 nm.

### Migration Assay

The HUVECs were seeded in a culture-insert (ibidi culture-insert 2 well, ibidi GmbH, Martinsried, Germany). After cultured 24 h, cells were transfected with miR-21 mimic (5 nM), inhibitor (10 nM), their respective negative control will be maintained in complete growth medium for 24 h. Furthermore, cells were removed the culture-insert and washed the cells with PBS to remove non-adherent cells. The culture was continued with normoxia or hypoxia (3% oxygen; BioSpherix Medical) and was be set as 0 h. The same wound areas were observed and photographed under a light microscope (Olympus BX51, Olympus Optical Co., Ltd., Tokyo, Japan). The distance of the scratch closure was examined at 0 and 24 h and then quantified the areas using imageJ analysis.

### Tube Formation Assay

The tube formation of HUVECs were evaluated by Matrigel assay. HUVECs were pretreated with miR-21 mimic (5 nM), inhibitor (10 nM) for 24 h. After treatment, HUVECs were seeded on Matrigel-coated 24-well plates (CORNING, Thermo Fisher Scientific, CA, United States). Tube formation was quantified by counting the number of branch points and calculating the total tube length in five randomly chosen fields from each well.

### Western Blotting

Cultured cell and muscle tissues were homogenized in lysis buffer, the supernatant was collected and its protein content was measured by Pierce™ BCA protein assay kit (Thermo Fisher Scientific, CA, United States). Equal amounts of protein (50 μg/lane) were separated in SDS-PAGE and blocked with 5% milk room temperature for 1 h. After blocking, the membranes were incubated with primary antibodies and corresponding peroxidase-conjugated secondary antibodies (Jackson ImmunoResearch Laboratories Inc., West Grove, PA, United States). Primary antibodies against phosphatase and tensin homolog (PTEN) (1:1,000, St John’s Laboratory, London, United Kingdom), Protein kinase B (AKT) (1:1,000, Cell Signaling, Massachusetts, United States), Phosphoinositide 3-kinases (PI3K) (1:1,000; Abcam, Cambridge, MA, United States), Nuclear factor kappa B (NFκB) (1:1,000; Abcam, Cambridge, MA, United States), vascular endothelial growth factor (VEGF) (1:1,000, Merck Millipore, Billerica, MA, United States), BCL2 associated X (Bax), cleaved caspase 3 (1:500, Cell Signaling, Massachusetts, United States), matrix metallopeptidase 9 (MMP9), BCL2 associated agonist of cell death (BCL2) associated agonist of cell death Bad, Bcl-2 (1:1,000, Arigo Hsinchu, Taiwan, ROC), or GAPDH (1:5,000, Sigma-Aldrich Co., St Louis, MO, United States). Western blot image was obtained by ECL-Western blotting system (AVEGENE CHEMX 400). The expression of protein was normalized to GAPDH and quantified by Image J software (Bethesda, NIH, United States).

### A Terminal Deoxynucleotidyl Transferase-Mediated dUTP Nick End Labeling

Apoptotic cells in HUVECs were identified by Transferase-Mediated dUTP Nick End Labeling (TUNEL) staining according to the manufacturer’s protocol (BioVision, Milpitas, CA, United States). DAPI staining was used to count the total number of nuclei. The imaged were captured by a fluorescence microscope (Olympus BX51, Olympus Optical Co., Ltd., Tokyo, Japan) and apoptotic HUVECs were quantified and classified as TUNEL + cells. The results are presented as the ratio of positive to total cells.

### Patients and Clinical Study Design

We prospectively included patients preparing for percutaneous transluminal angioplasty (PTA) for PAD in Chi-Mei Medical Center. Before interventions, the sera and clinical information were collected. Blood pressures and heart rates were measured before PTA. Patients who were lost to follow-up, preparing for amputation, in a status of active infection or with an expected lifespan less than 1 year were excluded. The endpoint of major adverse limb events (MALE) was defined as reintervention on the index arterial segment or amputation of the index limb. The median follow-up duration was 20 months (interquartile range, IQR: 12–38 months). The study was conducted in strict accordance with the Declaration of Helsinki on Biomedical Research involving human subjects and was approved by the local ethics committee (IRB: 10307-003). Additionally, this study is registered in Clinical Trials (protocol ID: CMMC10705-003).

### Statistical Analysis

The chi-squared tests were used to compare differences in miR-21 and comorbidity frequencies between PAD patients with and without MALEs. After testing for normality, continuous variables were compared between young and aged PAD patients using the Mann-Whitney *U*-test. The Kaplan-Meier method was used to plot MALEs, and group differences were compared *via* the log-rank test. The hazard ratio (HR) of MALEs was estimated using the Cox proportional hazard regression model adjusted for the potential confounding factors and comorbidities. A two-tailed *P*-value < 0.05 was considered statistically significant for all of the tests. All of the analyses were conducted using SAS software version 9.4 (SAS Institute, Cary, NC, United States).

## Results

### MicroRNA-21 Promotes Perfusion Recovery in the Hind Limb Ischemia Model

**FIGURE 1:**
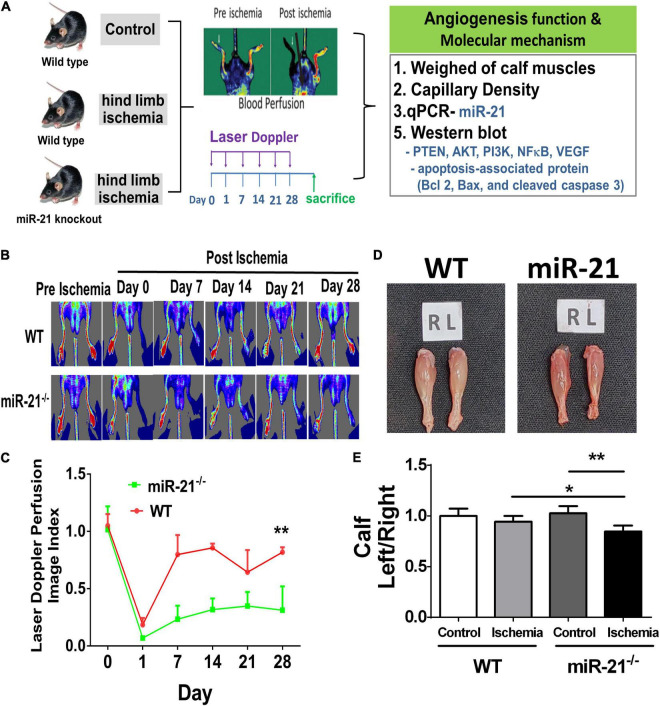
Impaired angiogenesis of wild-type and microRNA-21 (miR-21) ^– /–^ mice post hindlimb ischemia surgery. **(A)** The study design of limb ischemia in wild-type and microRNA-21 (miR-21) ^– /–^ mice. **(B)** Representative images of laser Doppler perfusion flow in limb ischemia in mice. **(C)** Quantification of LDPI at 0, 7, 14, 21, and 28 days after surgery and recovery of perfusion as expressed by the blood flow ratio (ischemic leg/non-ischemic leg) were measured. **(D)** Representative images and weight quantifications of harvested ischemic (left; L) limbs compared with non-ischemic (right; R) limbs. **(E)** The graph quantifying calf muscle weight loss in WT and miR-21^– /–^. *N* = 6, *P < 0.05, **P < 0.01.

To investigate the effect of miR-21 on tissue revascularization after ischemic insult, we established hind limb ischemia in WT and miR-21^–/–^ mice (Figure 1A). Using RT-qPCR, the present study revealed that the miR-21 expression levels in the muscle from ischemic hind limb of WT were significantly higher compared with the non-ischemic limb 28 days after surgery (Supplementary Figure 1). After surgery, hind limb blood flow was monitored for up to 28 days by LDPI (Figure 1B). At 28 days, recovery of limb perfusion was significantly increased in the WT mice compared with the miR-21^–/–^ mice (Figure 1C), and calf weight loss was attenuated (Figures 1D,E).

### MicroRNA-21 Increases Angiogenesis and Inhibits Apoptosis Associated With the Phosphatase and Tensin Homolog/Protein Kinase B Pathway in a Mouse Model of Hindlimb Ischemia

**FIGURE 2:**
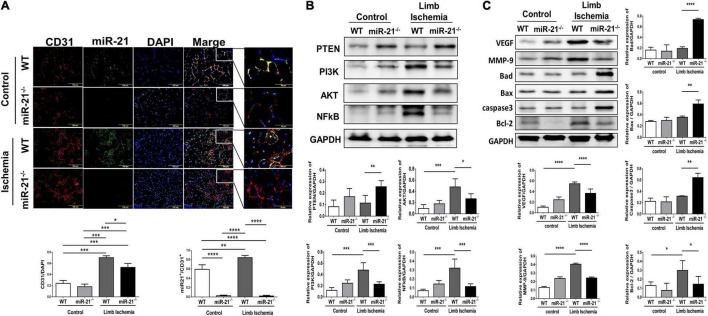
Impaired capillary density, expression of angiogenesis-, proliferation-, and apoptosis-associated proteins in wild-type and microRNA-21 (miR-21) ^– /–^ mice post hindlimb ischemia surgery. **(A)** Representative images and quantification of CD31 immunostaining representing capillary density. miR-21 *in situ* hybridization indicated that miR-21 was mainly expressed in endothelial cells in wild-type mice after limb ischemic surgery. **(B)** Representative blots and quantification of PTEN, PI3K, AKT, NFκB, VEGF, MMP9 and **(C)** apoptosis-associated protein expression in the ischemic limbs of wild-type and miR-21^– /–^ mice. The experiment was repeated in triplicate, *P < 0.05, **P < 0.01, and ***P = 0.001, and ****P < 0.001.

After hindlimb ischemia surgery, the expression of CD31, representing capillary density, was increased in ischemic calf muscle of WT and miR-21^–/–^ mice. However, the CD31 density in the ischemic calf muscle of WT mice was higher than that of miR-21^–/–^ mice (Figure 2A). To identify the main cells that present high expression of miR-21, using a miR-21 oligonucleotide probe, we co-stained miR-21 with CD31. Interestingly, we observed that the major expression of miR-21 located in endothelial cells. Also, endothelial expression of miR-21 significantly increased in mice post the surgery of limb ischemia. To further investigate the potential targets of miR-21, a recent study demonstrated that through suppressing PTEN, miR-21 could activate cell proliferation and migration (15). Additionally, PTEN has been shown to downregulate the PI3K/Akt/VEGF signaling pathway and constitutes a major determinant of neovascularization at ischemic sites (16, 17). Using Western blotting, the expression levels of PTEN, PI3K, AKT, and NFκB were measured in the hind limb muscles. Interestingly, we revealed that abolishing miR-21 significantly upregulated PTEN expression but downregulated PI3K, AKT, and NFκB expression, especially under limb ischemic conditions (Figure 2B). Additionally, angiogenesis-associated proteins, including VEGF and MMP9, were decreased in miR-21^–/–^ mice with hind limb ischemia (Figure 2C). Compared with WT mice, the expression of proapoptotic proteins, such as Bad, Bax, and cleaved caspase 3, was elevated, but the expression of the antiapoptotic protein Bcl-2 was decreased in miR-21^–/–^ mice with hind limb ischemia.

### MicroRNA-21 Enhances Angiogenic Capability in Human Umbilical Vein Endothelial Cells Under Hypoxic Stimuli

**FIGURE 3:**
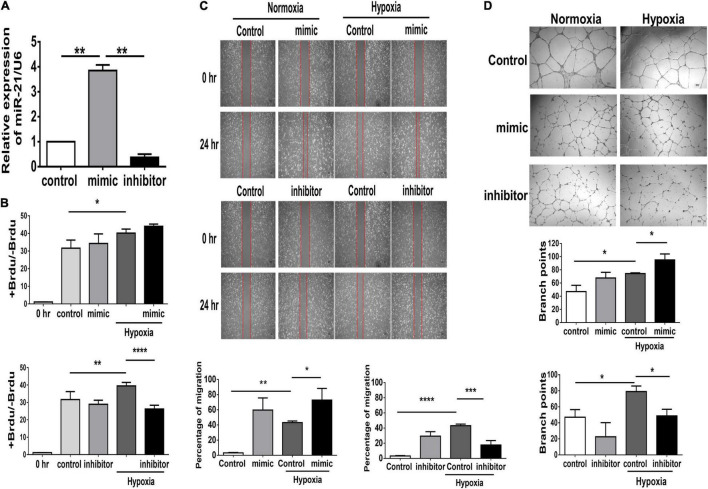
Suppression of miR-21 attenuates proliferation, migration and tube formation in human umbilical vein endothelial cells (HUVECs). HUVECs were transfected with miR-21 mimic or inhibitor for 24 h before hypoxia stimulation. **(A)** The intracellular expression of miR-21 in HUVECs was determined by RT–qPCR. **(B)** Cell proliferation, evaluated by measuring BrdU incorporation into cells, **(C)** cell migration after 24 h, **(D)** tube formation, evaluated by the branched points in HUVECs pretreated with miR-21 mimic or inhibitor under hypoxia stimulation for 24 h. The experiment was conducted in triplicate, *P < 0.05, **P < 0.01, and ***P = 0.001, and ****P < 0.001.

In PAD, angiogenesis is a major process involved in repairing the microvasculature in hind limb ischemia. Our *in vivo* study found that deletion of miR-21 impaired the recovery of limb perfusion and decreased capillary density in mice that received hind limb ischemia. Furthermore, using microRNA mimics in HUVECs, we investigated whether overexpression of miR-21 could improve angiogenesis and perfusion recovery following hindlimb ischemia (Supplementary Figure 2A). The miR-21 mimic and inhibitor showed sufficient effects on the overexpression and knockdown of miR-21 in HUVECs compared with the negative control (Supplementary Figure 2B and Figure 3A). Under hypoxic conditions, there was a significant increase in cell proliferation in HUVECs transfected with the miR-21 mimic compared with HUVECs transfected with the vehicle control, whereas the miR-21 inhibitor decreased cell proliferation (Figure 3B). Likewise, transfection with miR-21 mimic markedly improved cell migration and tube formation in HUVECs under hypoxic conditions compared with those transfected with vehicle control (Figures 3C,D). Conversely, by suppressing miR-21 expression using a miR-21 inhibitor, we observed a significant attenuation of the migration and tube formation capabilities of HUVECs under hypoxia.

### MicroRNA-21 Increases Angiogenesis and Inhibits Apoptosis in Human Umbilical Vein Endothelial Cells Under Hypoxic Stimuli *via* the Phosphatase and Tensin Homolog/Phosphoinositide 3-Kinases/Protein Kinase B Pathway

**FIGURE 4:**
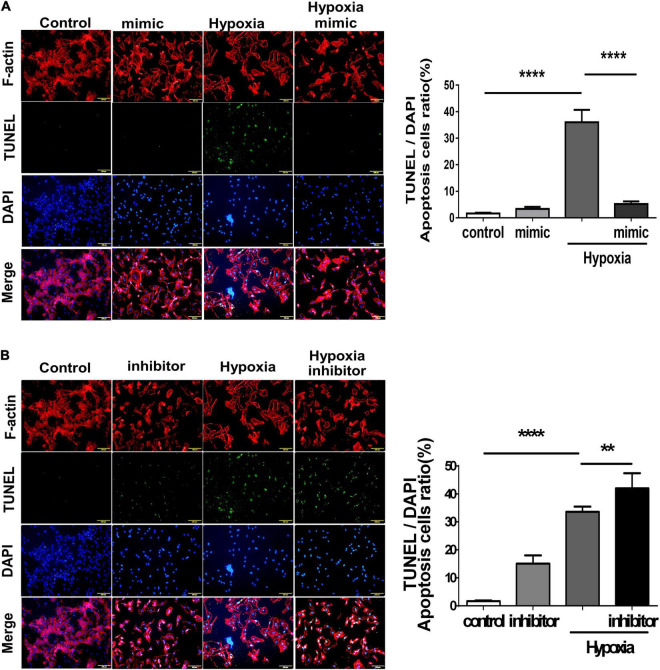
Overexpression of miR-21 mitigates hypoxia-induced cell apoptosis in human umbilical vein endothelial cells (HUVECs). TUNEL staining in HUVECs pretreated with **(A)** miR-21 mimic or **(B)** miR-21 inhibitor under hypoxia stimulation for 24 h. The experiment was conducted in triplicate, **P < 0.01 and ****P < 0.001.

**FIGURE 5:**
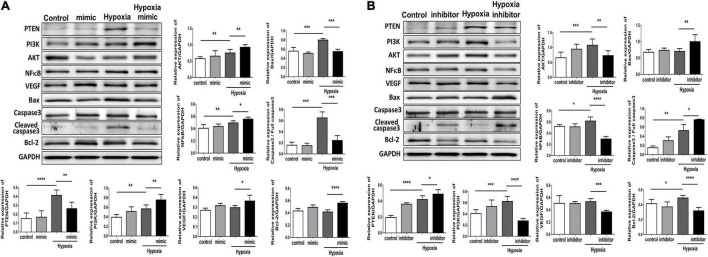
Overexpression of miR-21 augments angiogenesis and proliferation and associated proteins but attenuates apoptosis-associated proteins in human umbilical vein endothelial cells (HUVECs) after hypoxia stimulation. Representative blots and quantification of PTEN, PI3K, AKT, NFκB, VEGF, MMP9, and apoptosis-associated protein expression in HUVECs pretreated with **(A)** miR-21 mimic or **(B)** miR-21 inhibitor under normoxia or hypoxia for 24 h. The experiment was repeated in triplicate, *P < 0.05, **P < 0.01, and ***P = 0.001, and ****P < 0.001.

Using TUNEL staining, we measured the degree of apoptosis in HUVECs under hypoxic stimuli. Notably, overexpression of miR-21 in HUVECs inhibited hypoxia-mitigated cell apoptosis. Conversely, the suppression of miR-21 partially exacerbated hypoxia-induced cell apoptosis (Figure 4). To further investigate the angiogenetic and antiapoptotic mechanism of miR-21 in HUVECs under hypoxic stimuli, we used a miR-21 mimic and found that overexpression of miR-21 significantly inhibited the expression of PTEN but increased the expression of PI3K, AKT, NFκB, and VEGF in HUVECs under hypoxic stimuli (Figure 5A). Additionally, the apoptosis-associated proteins Bad, Bax, and cleaved caspase 3 were decreased, but the antiapoptotic protein Bcl-2 was increased in HUVECs transfected with miR-21 under hypoxic stimuli. In contrast, the above results were reversed in HUVECs transfected with miR-21 inhibitor under hypoxic stimuli (Figure 5B). Taken together, the results indicated that miR-21 expression was associated with angiogenesis and apoptosis in HUVECs under hypoxic stimuli through mediating the PTEN/PI3K/AKT pathways.

### Low Levels of Circulating MicroRNA-21 Were Associated With More Major Adverse Limb Events

**FIGURE 6:**
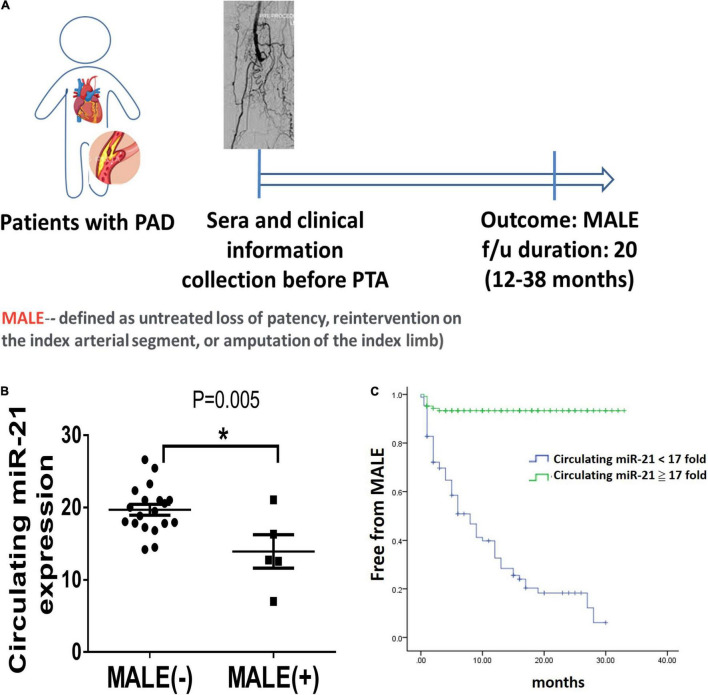
Lower circulating microRNA-21 (miR-21) expression in patients with peripheral artery disease (PAD) is associated with more major adverse limb events (MALEs). **(A)** The design of a clinical study focusing on patients with PAD. **(B)** The expression of circulating miR-21 in patients with or without the development of MALEs. **(C)** A Kaplan–Meier plot of MALEs among patients with high or low expression of circulating miR-21 (*N* = 20, *P < 0.05). *The cutoff value of miR-21 was defined as a 17-fold change compared with the control expression.

To further evaluate whether miR-21 reflects the development of MALEs in patients with PADAs, we further measure the expression of circulating miR-21 in those patients before PTA. As the study design illustrated in Figure 6A, after excluding 14 patients who failed to meet the inclusion criteria, this study prospectively enrolled 216 patients receiving PTA for PAD, 74 of whom subsequently developed MALEs (Supplementary Table 1). Compared with patients free from MALEs, despite no significant differences in age and sex, those developing MALEs had more coronary artery disease (CAD), heart failure (HF) or chronic kidney disease (CKD) but less hyperlipidemia or previous stroke. Most of the studied patients received either anti-platelet agents, anti-coagulants or even combination therapies. One third of patients prescribed with statins. There was no significant difference in term of cardiovascular drugs between the two groups. Notably, the levels of circulating miR-21 were significantly lower in patients developing MALEs than in those free from MALEs (12.88 ± 4.52 ng/ml vs. 19.58 ± 3.08 ng/ml, *p* = 0.001) (Figure 6B). In multivariable Cox regression analysis, we found that a history of HF (HR: 1.97, CI: 1.06–3.64, *p* = 0.03) and the expression of circulating miR-21 (HR: 0.83, CI: 0.79–0.87, *p* = 0.001) were significantly associated with the occurrence of MALEs (Supplementary Table 2). Furthermore, using the cutoff value of 17-fold change of circulating miR-21, it is still a sensitive biomarker to predict a lower risk of MALEs in patients with PAD (HR: 0.07, CI: 0.03–0.15, *p* = 0.001). The Kaplan–Meier plot also showed that circulating miR-21 at a 17-fold change was associated with a significantly lower probability of being free from MALEs (Figure 6C). Taken together, our findings showed that miR-21 could not only be a biomarker predicting the subsequent MALEs but is associated with the ability of angiogenesis.

## Discussion

**FIGURE 7:**
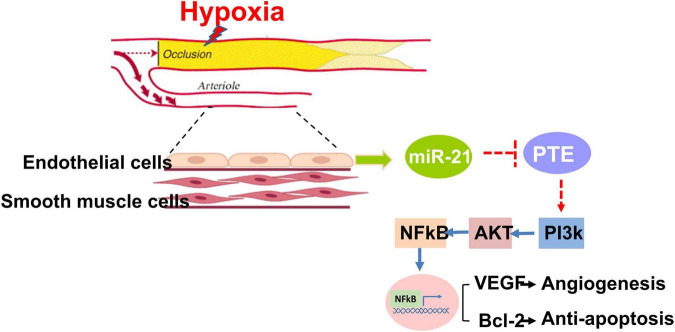
Summary of miR-21 in augmenting hypoxia-triggered angiogenesis and attenuating apoptosis in the process of limb ischemia.

In this study, we found that among patients with PAD, high expression of circulating miR-21 was associated with fewer MALEs, including limb loss or repeated interventions. Mechanistically, compared with wild-type mice, the capability of angiogenesis was significantly attenuated in miR-21^–/–^ mice following 28 days of hindlimb ischemic surgeries. Likewise, under hypoxia, proliferation, migration, and tube formation were significantly impaired in HUVECs treated with the miR-21 inhibitor but rescued by the miR-21 mimic. Our *in vitro* study also suggested that by regulating the PTEN/PI3K/AKT pathways, miR-21 could suppress hypoxia-triggered apoptosis in HUVECs. Collectively, miR-21 could not only be a biomarker reflecting the development of MALEs in patients with PAD but also be mechanistically associated with proliferation, migration and tube formation in endothelial cells under hypoxia. A summary of our findings is illustrated in Figure 7.

With its increasing prevalence, PAD, a new threat to public health beyond coronary artery disease, involves aberrant vascular endothelial cell proliferation and angiogenesis (2, 7, 18). Patients with PAD may develop MALE during the late stage of disease propagation, which markedly reduces blood flow to the extremities and progresses to the point of severe resting pain and even tissue loss (2, 18). Although surgical or endovascular revascularization has been used for the treatment of PAD, some patients fail to tolerate these intricate and high-risk surgeries (2). Thus, discovering a pharmacological therapy is crucial in the management of PAD. Additionally, in patients with PAD, biomarkers facilitate physicians in identifying high-risk populations who may have poor outcomes in limb salvage (19). Herein, among 216 patients receiving PTA for PAD, we observed that low expression of circulating miR-21 was significantly associated with subsequent MALEs. Furthermore, using the cutoff value of 17-fold change of circulating miR-21, it could be a sensitive biomarker to differentiate the development of MALEs in patients with PAD. Our study, using a dataset from bench to bedside, demonstrates some potential underlying physiological pathways in terms of the application of miR-21 in PAD, which could be taken into accounts in clinical uses.

In addition to anti-thrombosis and anti-atherosclerosis, current biologic therapies have focused on the potential to promote tissue angiogenesis in limb musculature (7). Among the pro- and antiangiogenic factors, miRNAs regulate a wide range of physiological and pathological processes, including cell development, cell metabolism, cell aging, cell death, and other key biological processes (9). According to recent findings, miRNAs are abundant in the vascular system and play crucial roles in tissue recovery and angiogenesis (20, 21). Previous evidence has demonstrated that the expression of miRNAs is significantly altered under hypoxic conditions (22, 23). Among multiple miRNAs, miR−21 is consistently induced in response to hypoxia. It is also known to promote angiogenesis and to suppress apoptosis (24–26). A study by Sabatel et al. demonstrated that miR-21 overexpression reduced endothelial cell proliferation, migration and the ability of these cells to form tubes, while the inhibition of miR-21 using an LNA-anti-miR led to opposite effects (27). The results from another study demonstrated that miRNA-21 exhibits an antiangiogenic function by decreasing apoptosis and activating the nitric oxide pathway (28). However, most of those studies were limited in cellular observations, and whether miR-21 could promote angiogenesis in animal models of hindlimb ischemia requires more evidence.

The regulation of miRNAs expression could be mediate parallelly by physical and chemical stimuli. In response to hypoxia stress, cells alter DNA transcription, which induces hypoxia-inducible factor-1 (HIF-1). HIF-1 is a major transcription factor that promotes ischemia-driven angiogenesis (29). Previous studies indicated that several miRNAs were associated with the hypoxia induced upregulation of HIF-1 (30, 31). For example, miR-155 were upregulated by HIF-1α under hypoxia (32). Also, HIF-1α induced miR-210 expression in HUVEC results in increased tubulogenesis and increased VEGF-induced cell migration (33). HIF-1α also regulated miRNA expression *via* the modulation of their transcription factors. Liu et al. report found that HIF-1α increased miR-21 expression by directly binding to the region of miR-21 promote under hypoxic conditions (34). Additionally, hypoxia also mediated important post-translational modifications, Drosha, and Dicer that modulate the activity of proteins involved in the biogenesis and functionality of miRNAs (30). In term of another major contributor to PAD, diabetes has been found to tightly associated with circulating miR-21 expression (35). Also, the intervention of cardiovascular drugs such as statins, may suppress the expression of miR-21 in cancer cells but the effects of statins on miR-21 associated angiogenesis in PAD requires further investigations (36).

Using a rat model of hindlimb ischemia, we found that abolishing miR-21 resulted in an impairment of angiogenesis. In terms of the targets of miR-21, Meng et al. reported that it directly regulates the expression of *PTEN*, a multifunctional tumor suppressor gene, in human hepatocellular cancer (37). Notably, *PTEN* regulates cell proliferation, migration, adhesion, and angiogenesis and can be found in almost all tissues in the body (15, 16). In a model of acute kidney injury, Song et al. also observed that miR-21 protects against ischemia/reperfusion injury by preventing epithelial cell apoptosis through regulating the PTEN/AKT/mTOR/HIF pathways (22). Likewise, by overexpressing miR-21 in human stem cells, Zhou et al. improved neovascularization in chronic limb ischemia by enhancing HIF-1α activity (24). In this translational study, we also demonstrated that by targeting PTEN, miR-21 activated the PI3K/AKT signaling pathways, suppressed apoptosis and promoted angiogenesis in limb ischemia. Additionally, previous studies showed that miR-21 mediated PTEN expression leads to the upregulated NFkB expression (38–40). NFκB has been reported to mediate proliferation of vascular smooth muscle cells and is necessary for capillary tube formation (40). The activation of NFκB in endothelial cell leads to the expression of angiogenic factors such as VEGF (41). However, the direct contribution of NFkB to the downstream VEGF and Bcl-2 pathways is yet elucidated in this study. In the further, it will be interesting to know if the NFkB inhibition could exert similar effects of miR-21 inhibition on angiogenesis (40).

There are some limitations of this study. First, although we herein focused on miR-21, there should be other microRNAs involved in the process of PAD. Whole-miRNA transcriptome profiling performed in peripheral blood from patients with PAD and controls showed a 12-miRNA PAD-specific signature (20). Our findings only highlight the potential role of miR-21 in PAD, while further investigation, especially miR-21-based interventional studies, is required. Second, diabetes, obesity, atherosclerosis, and high blood pressure are known risk factors of PAD (42, 43). Previous studies investigated the therapeutic strategies on neovascularization in diabetic mice with limb ischemia (42, 43). Differently, herein we focused on studying the effect of miR-21 on endothelial cells in response to hypoxia. We found that miR-21 activated the PI3K/AKT signaling pathways by targeting PTEN resulting in suppressing apoptosis and promoting angiogenesis in limb ischemia. However, the regulatory role of miR-21 in improving the angiogenesis in other chronic diseases such as diabetes with PAD remains to be further clarified. Third, given that the microRNAs for miR-21 measurements were derived from whole calf tissues, whether miR-21 expression was from vessels (endothelial cells) or other cells is uncertain. To note, using *in situ* hybridization, we observed co-expressions of miR-21 and CD31, which implied that miR-21 was mainly expressed in endothelial cells in this hindlimb ischemic model. Last, the blood vessels can grow either *via* the process of angiogenesis or arteriogenesis (43). Through sprouting new endothelial cells, angiogenesis is defined as the formation of new blood vessels from existing ones. Under hypoxia, HIF-1 activated transcription of angiogenic growth factors including VEGF, fibroblast growth factors (FGF), and MMP to facilitate angiogenesis (43, 44). On the other hand, arteriogenesis is the remodeling of preexisting collateral arteries to generate larger conductance vessels to replace the existing ones (43, 44). With an increasing shear stress, arteriogenesis leads to the upregulation of cell adhesion molecules including intercellular adhesion molecule (ICAM), vascular cell adhesion molecule (VCAM), and selectins (43). Nevertheless, given that we mainly focused on the effect of miR-21 on angiogenesis in responses to hypoxia, the involvement of arteriogenesis in this study requires further investigations. In addition to endothelial cells, vascular smooth muscle, myoblasts, and macrophage polarization should also be considered in the process of angiogenesis in PAD (45, 46). Further studies will be required to investigate the effect of miR-21 on the recruitment, polarization and inflammatory phenotypes of macrophages and especially the crosstalk between cells, focusing on how it promotes angiogenesis.”

## Conclusion

Collectively, our findings showed that miR-21 could be a pivotal regulator of cell apoptosis and angiogenesis post limb ischemia. Not only as a biomarker but also a potential therapeutic target, miR-21 was found to promote proliferation, migration and tube formation in endothelial cells and mitigate limb ischemia. The translation of miR-21 to the clinical arena for the detection and management of clinical PAD represents significant promise.

## Data Availability Statement

The original contributions presented in the study are included in the article/Supplementary Material, further inquiries can be directed to the corresponding author/s.

## Ethics Statement

The studies involving human participants were reviewed and approved by IRB: 10307-003. The patients/participants provided their written informed consent to participate in this study. The animal study was reviewed and approved by Chi-Mei Medical Center.

## Author Contributions

W-TC and Y-WL: conceptualization, methodology, validation, data curation, formal analysis, writing—original draft preparation, writing—review and editing, visualization, project administration, and funding acquisition. Z-CC, J-YS, and C-SH: software. W-TC, Y-WL, P-SH, Y-CL, S-YT, T-HC, Z-CC, J-YS, and C-SH: investigation. P-SH, Y-CL, S-YT, T-HC, Z-CC, J-YS, and C-SH: resources. T-HC, Z-CC, J-YS, and C-SH: supervision. All authors have read and agreed to the published version of the manuscript.

## Conflict of Interest

The authors declare that the research was conducted in the absence of any commercial or financial relationships that could be construed as a potential conflict of interest.

## Publisher’s Note

All claims expressed in this article are solely those of the authors and do not necessarily represent those of their affiliated organizations, or those of the publisher, the editors and the reviewers. Any product that may be evaluated in this article, or claim that may be made by its manufacturer, is not guaranteed or endorsed by the publisher.

## Supplementary Material

Supplementary Figure 1 The expression of miR-21 in calf tissue in wild-type and mir-21-/- mice after limb ischemic surgery.

Supplementary Figure 2 **(A)** The study design of *in vitro* hypoxia in HUVECs mimicking limb ischemia. **(B)** The expression of miR-21 in HUVCs treated with miR-21 mimic or inhibitor at the indicated concentration.
