# Supplementary figures and images for: Deletion of MicroRNA-21 Impairs Neovascularization Following Limb Ischemia: From Bedside to Bench
Source: Front Cardiovasc Med. 2022 Apr 26;9:826478. doi: 10.3389/fcvm.2022.826478 (PMC9086398; doi:10.3389/fcvm.2022.826478)

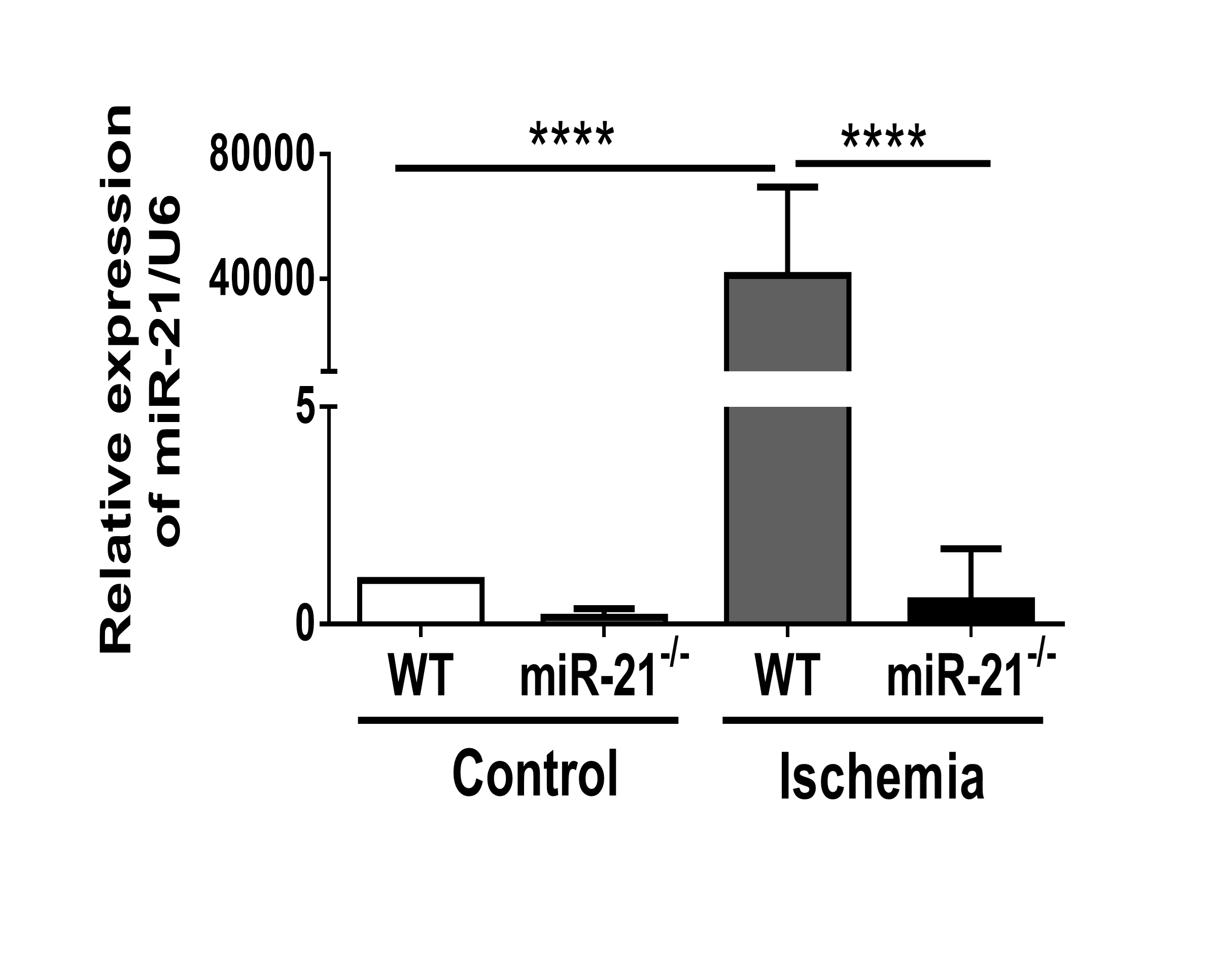

Supplement: Supplementary Figure 1 — The expression of miR-21 in calf tissue in wild-type and mir-21-/- mice after limb ischemic surgery. [file Image_1.TIF]

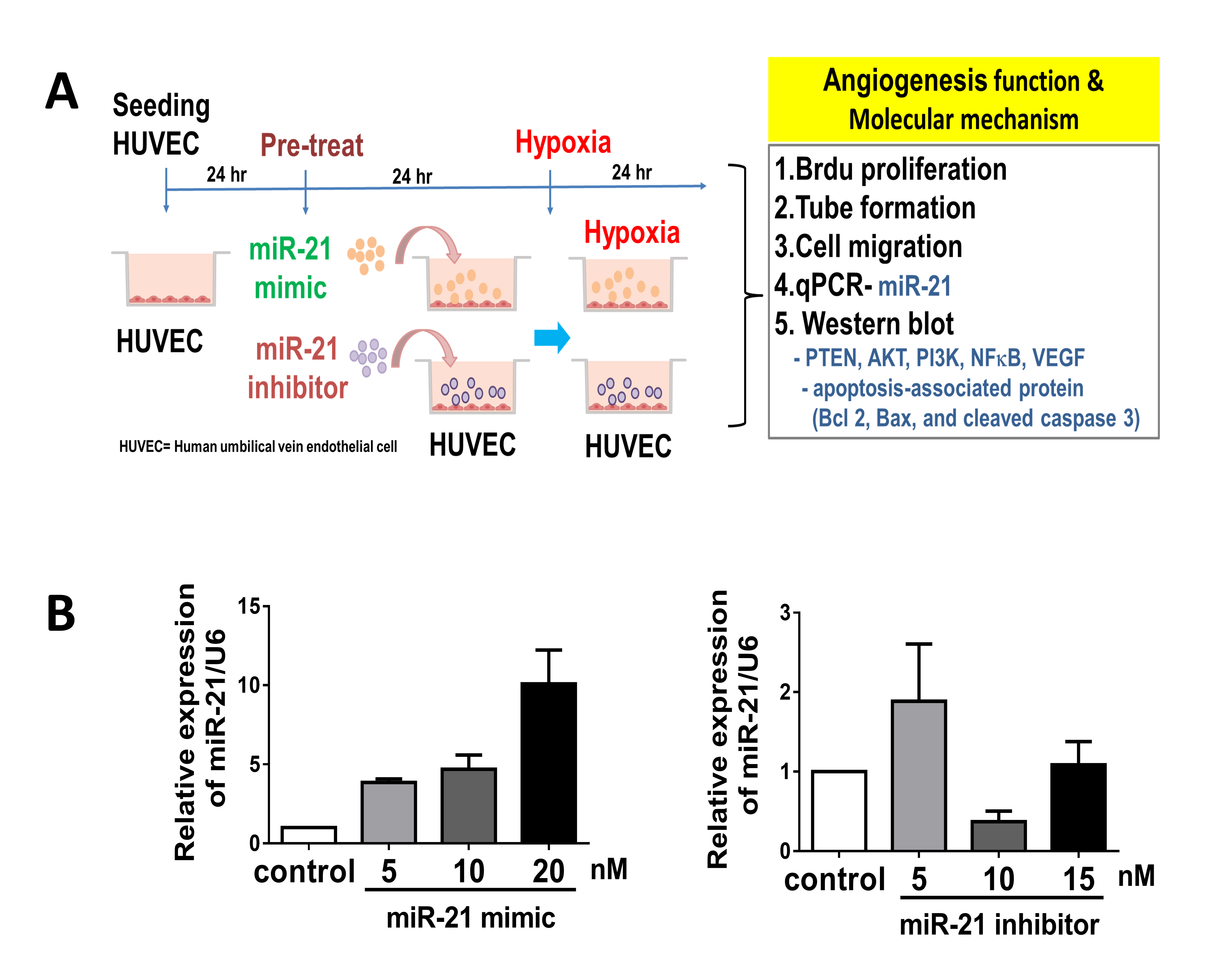

Supplement: Supplementary Figure 2 — (A) The study design of in vitro hypoxia in HUVECs mimicking limb ischemia. (B) The expression of miR-21 in HUVCs treated with miR-21 mimic or inhibitor at the indicated concentration. [file Image_2.TIF]
