# Supplementary material for: Deletion of MicroRNA-21 Impairs Neovascularization Following Limb Ischemia: From Bedside to Bench
Source: Front Cardiovasc Med. 2022 Apr 26;9:826478. doi: 10.3389/fcvm.2022.826478 (PMC9086398; doi:10.3389/fcvm.2022.826478)
Supplement: Supplementary file 3 [file Table_1.DOCX]

Supplemental table 1. The baseline characteristics of patients with peripheral arterial disease (N=216)

| **Factors** | **MALE (-) (N=142)** | **MALE (+) (N=74)** | **P value** |
| --- | --- | --- | --- |
| Age (y/o) | 67.53±13.16 | 70.13±11.52 | 0.15 |
| Gender, n (%) | 92 (64.78) | 47(63.51) | 0.88 |
| BMI (kg/M^2^) | 24.71±4.45 | 24.62±4.74 | 0.89 |
| Heart rate (bpm) | 81.74±18.08 | 81.01±14.92 | 0.76 |
| SBP (mmHg) | 151.73±30.21 | 155.47±27.12 | 0.37 |
| DBP (mmHg) | 77.21±15.36 | 77.63±16.2 | 0.85 |
| Smoking, n (%) | 93(65.49) | 44(59.45) | 0.43 |
| **Medical history** | | | |
| Diabetes, n (%) | 80(56.33) | 50(67.56) | 0.12 |
| Hypertension, n (%) | 100(70.42) | 48(64.86) | 0.07 |
| CAD, n (%) | 57(40.14) | 39(52.71) | 0.03 |
| Heart failure, n (%) | 13(9.15) | 14(18.91) | 0.05 |
| Hyperlipidemia, n (%) | 66(46.47) | 22(29.72) | 0.02 |
| Previous stroke, n (%) | 26(18.31) | 5(6.75) | 0.02 |
| Cancer, n (%) | 13(9.154) | 3(4.05) | 0.27 |
| CKD (including H/D), n (%) | 26(18.31) | 23(31.08) | 0.03 |
| **Laboratory data** | | | |
| eGFR(ml/min/1.73m^2^) | 60.62±33.18 | 51.71±32.25 | 0.07 |
| ALT(mg/dl) | 25.81±23.26 | 24.82±35.97 | 0.82 |
| Total Cholesterol(mg/dl) | 153.51±37.05 | 152.68±58.11 | 0.92 |
| LDL(mg/dl) | 89.32±36.82 | 92.77±53.84 | 0.7 |
| Triglyceride(mg/dl) | 170.54±191.01 | 183.55±318.5 | 0.79 |
| Circulating miR-21 | 19.58±3.08 | 12.88±4.52 | 0.001 |
| **Drug history** | | | |
| Anti-platelet agents, n (%) | 102(71.8) | 54(72.9) | 0.49 |
| Anti-coagulants, n (%) | 47(33) | 28(37.8) | 0.29 |
| Statins, n (%) | 49(34.5) | 20(27) | 0.05 |
| Anti-diabetic drugs, n (%) | 77(54.2) | 46(62.6) | 0.12 |

Data are n (%) or mean ± standard error.; *P <*0.05 as significance.

BMI= body mass index; SBP = systolic blood pressure; DBP = diastolic blood pressure; CAD= coronary artery disease; CKD= chronic kidney disease; eGFR= estimated Glomerular filtration rate; ALT= Alanine aminotransferase; LDL= low-density lipoprotein; miR-21= microRNA-21; MALE= major adverse limb events.
