# Supplementary material for: Deletion of MicroRNA-21 Impairs Neovascularization Following Limb Ischemia: From Bedside to Bench
Source: Front Cardiovasc Med. 2022 Apr 26;9:826478. doi: 10.3389/fcvm.2022.826478 (PMC9086398; doi:10.3389/fcvm.2022.826478)
Supplement: Supplementary file 4 [file Table_2.DOCX]

Supplemental table 2. The univariate and multivariable analysis of MALE in patients with peripheral arterial disease (PAD)

|  | **Univariate** | | **Multivariate** | | | |
| --- | --- | --- | --- | --- | --- | --- |
|  |  |  | Model 1 | | Model 2 | |
| Parameters | HR | p | HR | p | HR | p |
| Hypertension | 0.79(0.49-1.28) | 0.34 |  |  |  |  |
| CAD | 1.36(0.86-2.15) | 0.18 |  |  |  |  |
| Heart failure | 2.11(1.18-3.78) | 0.012 | 1.97(1.06-3.64) | 0.030 | 1.86(1.02-3.37) | 0.041 |
| Hyperlipidemia | 1.73(1.05-2.86) | 0.03 | 0.64(0.37-1.1) | 0.108 | 0.78(0.46-1.34) | 0.379 |
| Previous stroke | 3.02(1.21-7.49) | 0.017 | 0.5(0.19-1.27) | 0.149 | 0.49(0.19-1.24) | 0.137 |
| CKD (including H/D) | 1.95(1.18-3.2) | 0.008 | 1.46(0.85-2.5) | 0.160 | 1.43(0.84-2.42) | 0.179 |
| Circulating miR-21 | 0.82(0.78-0.86) | 0.001 | 0.83(0.79-0.87) | 0.001 |  |  |
| Circulating miR-21>17 fold changes | 0.06(0.03-0.13) | 0.001 |  |  | 0.07(0.03-0.15) | 0.001 |

Abbreviation as Table 1.

Model 1 including parameters of heart failure, hyperlipidemia, previous stroke, CKD (including H/D) and circulating miR-21 during follow-up while miR-21 as continuous variables; Model 2 including abovementioned parameters while miR-21 as categorical variables
